# Modulation of paclitaxel resistance by annexin IV in human cancer cell lines

**DOI:** 10.1054/bjoc.2000.1311

**Published:** 2000-06-02

**Authors:** E Kyu-Ho Han, S K Tahir, S P Cherian, N Collins, S-C Ng

**Affiliations:** Pharmaceutical Products Division, Cancer Research, Department 4N6, Building AP9, Abbott Laboratories, Abbott Park, IL, 60064-3500, USA

**Keywords:** annexin, paclitaxel, drug resistance, antimitotic

## Abstract

A recurring problem with cancer therapies is the development of drug resistance. While investigating the protein profile of cells resistant to a novel antimitotic compound (A204197), we discovered an increase in annexin IV expression. When we examined the annexin IV protein expression level in a paclitaxel-resistant cell line (H460/T800), we found that annexin IV was also overexpressed. Interestingly a closely related protein, annexin II, was not overexpressed in H460/T800 cells. Immunostaining with either annexin II or IV antibody revealed that annexin IV was primarily located in the nucleus of paclitaxel-resistant H460/T800 cells. Short-term treatment of H460 cells with 10 nMpaclitaxel for up to 4 days resulted in induction of annexin IV, but not annexin II expression. In addition, there was an increase in annexin IV staining in the nucleus starting at day 1. Furthermore, cells pretreated with 10 nMpaclitaxel for 4 days resulted in cells becoming ~fivefold more resistant to paclitaxel. Transfection of annexin IV cDNA into 293T cells revealed that there was a threefold increase in paclitaxel resistance. Thus our results indicate that annexin IV plays a role in paclitaxel resistance in this cell line and it is among one of the earliest proteins that is induced in cells in response to cytotoxic stress such as antimitotic drug treatment. © 2000 Cancer Research Campaign


British Journal of Cancer (2000) **83**(1), 83–88
© 2000 Cancer Research Campaign
doi: 10.1054/ bjoc.2000.1311, available online at http://www.idealibrary.com on IDEAL®

### Modulation of paclitaxel resistance by annexin IV in human cancer cell lines

**E Kyu-Ho Han*, SK Tahir*, SP Cherian, N Collins and S-C Ng**

Pharmaceutical Products Division, Cancer Research, Department 4N6, Building AP9, Abbott Laboratories, Abbott Park, IL 60064-3500, USA

**Summary** A recurring problem with cancer therapies is the development of drug resistance. While investigating the protein profile of cells resistant to a novel antimitotic compound (A204197), we discovered an increase in annexin IV expression. When we examined the annexin IV protein expression level in a paclitaxel-resistant cell line (H460/T800), we found that annexin IV was also overexpressed. Interestingly a closely related protein, annexin II, was not overexpressed in H460/T800 cells. Immunostaining with either annexin II or IV antibody revealed that annexin IV was primarily located in the nucleus of paclitaxel-resistant H460/T800 cells. Short-term treatment of H460 cells with 10 nM paclitaxel for up to 4 days resulted in induction of annexin IV, but not annexin II expression. In addition, there was an increase in annexin IV staining in the nucleus starting at day 1. Furthermore, cells pretreated with 10 nM paclitaxel for 4 days resulted in cells becoming ~fivefold more resistant to paclitaxel. Transfection of annexin IV cDNA into 293T cells revealed that there was a threefold increase in paclitaxel resistance. Thus our results indicate that annexin IV plays a role in paclitaxel resistance in this cell line and it is among one of the earliest proteins that is induced in cells in response to cytotoxic stress such as antimitotic drug treatment. © 2000 Cancer Research Campaign

**Keywords:** annexin; paclitaxel; drug resistance; antimitotic

Efficacy of paclitaxel in cancer patients has been hampered by the ability of cells to develop paclitaxel resistance (Rowinsky et al, 1992; Rowinsky and Donehower, 1995). Therefore if we understand the mechanism of paclitaxel resistance we will be more successful in the treatment of human cancer. It has been shown that several factors contribute to paclitaxel resistance in vitro:

1. P-glycoprotein (P-gp), an energy-dependent drug efflux pump shown to confer multidrug resistance (MDR) (Gottesman and Pastan, 1988, 1993; Lehnert, 1996; Germann, 1996)
2. alterations in β-tubulin isotypes (Haber et al, 1995; Ranganathan et al, 1988, 1996, 1998 Kavallaris et al, 1997, 1999)
3. mutations in tubulins (Cabral et al, 1981; Schibler and Cabral, 1986; Giannakakou et al, 1997)
4. up-regulation of caveolin-1 (Yang et al, 1998).

Paclitaxel also activates molecules that are involved in signal transduction pathways such as c-jun N-terminal kinase (Blagosklonny et al, 1995; Wolfson et al, 1997; Wang et al, 1998).

Annexins are a family of calcium-dependent phospholipid binding proteins. There are at least 13 annexin family members with a similar structure, characterized by the presence of four or eight repeats of a 70 amino acid motif and a variable N-terminal end (Smith and Moss, 1994; Dubois et al, 1996; Benz-and Hofmann, 1997). Although the roles of the annexins are not well understood, they have been involved in various biological processes including inhibition of phospholipase A2, anticoagulation, endocytosis, exocytosis, inhibition of calcium channels and protein kinase C activity (Smith and Moss, 1994). It was also previously shown that annexin II was elevated in doxorubicin-resistant small-cell lung cancer cell line (Cole et al, 1992). During the course of our antimitotic research, we found that annexin IV was overexpressed in A204197-resistant human colon cancer HCT-15 cells as determined by two-dimensional protein mapping as well as mass spectroscopic analysis. Therefore, we tested whether annexin IV or annexin II was also overexpressed in a paclitaxel-resistant cell line, H460/T800. To our surprise annexin IV, but not annexin II, was overexpressed in the paclitaxel-resistant H460 lung cancer cell line. To further confirm the role of annexin IV in drug resistance, annexin IV cDNA was transfected into 293T cells. Annexin IV transfectants were more resistant to paclitaxel as compared to the vector controls. Thus our study indicates that annexin IV plays a role in regulating paclitaxel resistance.

*Received 15 November 1999*
*Revised 25 January 2000*
*Accepted 27 January 2000*

*Correspondence to:* S-C Ng

### MATERIALS AND METHODS

### Cell culture

Lung cancer cell line NCI-H460 (hereafter referred to as H460) and colon cancer cell line HCT15 were obtained from the American Tissue Culture Collection (ATCC) H460 and HCT15 cells were grown and maintained in RPMI medium plus 10% fetal bovine serum (FBS) or 20% FBS respectively. Paclitaxel-resistant cell lines were derived from the H460 parental cell line. H460 cells were initially selected with $IC_{50}$ concentration of paclitaxel and thereafter paclitaxel concentration was doubled every 2–3 weeks until 800 nM paclitaxel resistance was obtained. HCT15/A204197 cells were selected with an antimitotic compound, A204197 in HCT15 parental cells. HCT15/A204197 cells were maintained in 200 nM A204197. Embryonic kidney 293T cells were maintained

* Authors contributed equally to this work in Dulbecco's modified Eagle medium (DMEM) + 10% FBS. Paclitaxel and all other chemicals were obtained from Sigma (St Louis, MO, USA). The discovery and characterization of A204197 will be described elsewhere. Cell cultures were maintained in a 37°C incubator with 5% carbon dioxide.

### Two-dimensional gel electrophoresis

Two-dimensional gel electrophoresis was performed according to the method of O'Farrell (1975) as follows: isoelectric focusing (IEF) was carried out in glass tubes of inner diameter 2.0 mm using 2% pH 4–8 ampholines (BDH from Hoefer Scientific Instruments, San Francisco, CA, USA) for 9600 volts-hours. One microgram of an IEF internal standard, tropomyosin, was added to each sample. This protein migrates as a doublet with lower polypeptide spot of MW 33 000 and pI 5.2; an arrowhead on the stained gel marks its position. The enclosed tube gel pH gradient plot for this set of ampholines was determined with a surface pH electrode. After equilibration for 10 min in Buffer 'O' (10% glycerol, 50 mM dithiothreitol (DTT), 2.3% sodium dodecyl sulphate (SDS) and 0.0625 M Tris, pH 6.8) the tube gels were sealed to the top of stacking gels on top of 10% acrylamide slab gels (0.75-mm-thick) and SDS slab gel electrophoresis carried out for about 4 h at 12.5 mA/gel. The following proteins (Sigma Chemicals Co., St Louis, MO, USA) were added as MW standards to a well in the arose which sealed the tube gel to the slab gel: myosin (220 000), phosphorylase A (94 000), catalase (60 000), actin (43 000), carbonic anhydrase (29 000) and lysozyme (14 000). These standards appear as horizontal lines on the Coomassie brilliant blue R-250 stained 10% acrylamide slab gels. The silver-stained gels were dried between sheets of cellophane with the acid edge to the left. After slab gel electrophoresis, duplicate gels were transferred to transfer buffer (12.5 mM Tris, pH 8.8, 86 mM glycine, 10% methanol) transblotted onto polyvinylidene fluoride (PVDF) membrane overnight at 200 mA and approximately 100 volts/two gels. The spot was removed and mass spectroscopic analysis was performed.

### Protein extraction and Western blot analysis

Protein extracts were prepared from exponentially growing cells as described (Han et al, 1995, 1996). Briefly, cells were collected and the cell pellets were resuspended in lysis buffer (20 mM Tris–HCl pH 7.4, 2 mM EGTA, 2 mM EDTA, 6 mM β-mercaptoethanol, 1% NP-40, 0.1% SDS and 10 mM sodium fluoride, plus the protease inhibitors aprotinin (10 μg $ml^{-1}$), leupeptin (10 μg $ml^{-1}$) and phenylmethylsulphonyl fluoride (PMSF, 1 mM)). This suspension was sonicated three times with a Sonifier Cell Disrupter (Branson Ultrsonics Co, Danbury, CT, USA). Cells were spun briefly and supernatants were collected for determination of protein concentration by the Bio-Rad assay (Bio-Rad, San Diego, CA, USA). For Western blotting, 20 μg of protein from the total cell lysates were fractionated by SDS polyacrylamide gel electrophoresis (SDS-PAGE). The proteins on these gels were then transferred to immobilon-P membranes (Millipore, Bedford, MA, USA), using transfer buffer (25 mM Tris, 190-mM glycine, 10% methanol). Membranes were blocked with blocking buffer (50 mM Tris, 200 mM sodium chloride, 0.2% Tween-20, 3% non-fat dry milk), and the membranes were then incubated with the indicated antibodies. Human annexin IV and annexin II (1:1000, Transduction Laboratories, Lexington, KY, USA) antibodies were used. After treatment with blocking buffer without 3% non-fat dry milk (washing buffer), a dilute solution (1:2000) of horseradish peroxidase linked anti-rabbit donkey serum (Amersham, Arlington Heights, IL, USA) was added. Membranes were then washed with washing buffer and immune detection was performed using the enhanced chemiluminescence (ECL) Western blotting detection system (Amersham, Arlington Heights, IL, USA).

### Immunohistochemistry

The medium was aspirated off and the cells were fixed in 10% formalin in phosphate-buffered saline (PBS) for 30 min at room temperature. Cells were washed once in PBS and incubated in blocking buffer (2% bovine serum albumin (BSA), 0.2% non-fat dry milk, 0.4% Triton X-100 in PBS) for 1 h at room temperature. The blocking buffer was aspirated off and the cells were incubated with either a mouse anti-annexin II or annexin IV IgG (diluted 1:4000 in block buffer) for 1 h at 37°C. The cells were washed 3 × for 5 min each time in was buffer (0.2% Tween-20 in PBS) and incubated with a goat anti-mouse Alexa 488 conjugated IgG (diluted 1:1000 in block buffer) for 0.5 h at 37°C. The cells were washed 3 × for 5 min each time in wash buffer, wet mounted with Citifluor and imaged with a BioRad MRC-1000 confocal microscope using a 60× objective.

### Determination of $IC_{50}$

Cytotoxicity assays were performed in 96-well microtitier plates using the colourimetric method as described (Skehan et al, 1990). To measure cell proliferation, the sulphorhodamine binding assay (SRB) was performed in the presence of paclitaxel, colchicine, or nocodazole ($10^{-1}$ to $10^{-11}$M) in 96-well plates containing cells at 60–70% confluency. Cells were cultured in the presence of above drugs for 48 h, after which time they were fixed with 10% trichloroacetic acid, stained with 0.4% SRB in 1% acetic acid, washed and dried. The remaining dye was solubilized in Tris buffers, and the plates were read at O.D. 490 nM for quantification. All $IC_{50}$ values are a representation of 2–3 independent experiments.

### Annexin IV cDNA transfection

Annexin IV cDNA was purchased from the ATCC (ATCC#525860, Rockville, MD, USA). The insert (~1.5 kb, *Eco*RI/Not fragment) was subcloned into a pcDNA 3.1 (+) expression vector (Invitrogen, Carlsbad, CA, USA) in the sense orientation. After subcloning into the pcDNA vector, a large-scale preparation was performed using Qiagen plasmid purification kit (Qiagen, Chatsworth, CA, USA). Transfection into 293 T-cells was performed using Lipofectamine Plus kit (Gibco-BRL, Grand Island, NY, USA). After transfection, cells were selected in the presence of 1 mg $ml^{-1}$ G418, colonies were isolated and expanded.

### RESULTS

### Two-dimensional gel mapping analysis of HCT15/A204197 cells

A204197 is a novel antimitotic compound that binds to the colchicine-binding site on tubulin (data to be published elsewhere). To understand the mechanism of A204197 resistance, we

 © 2000 Cancer Research Campaign

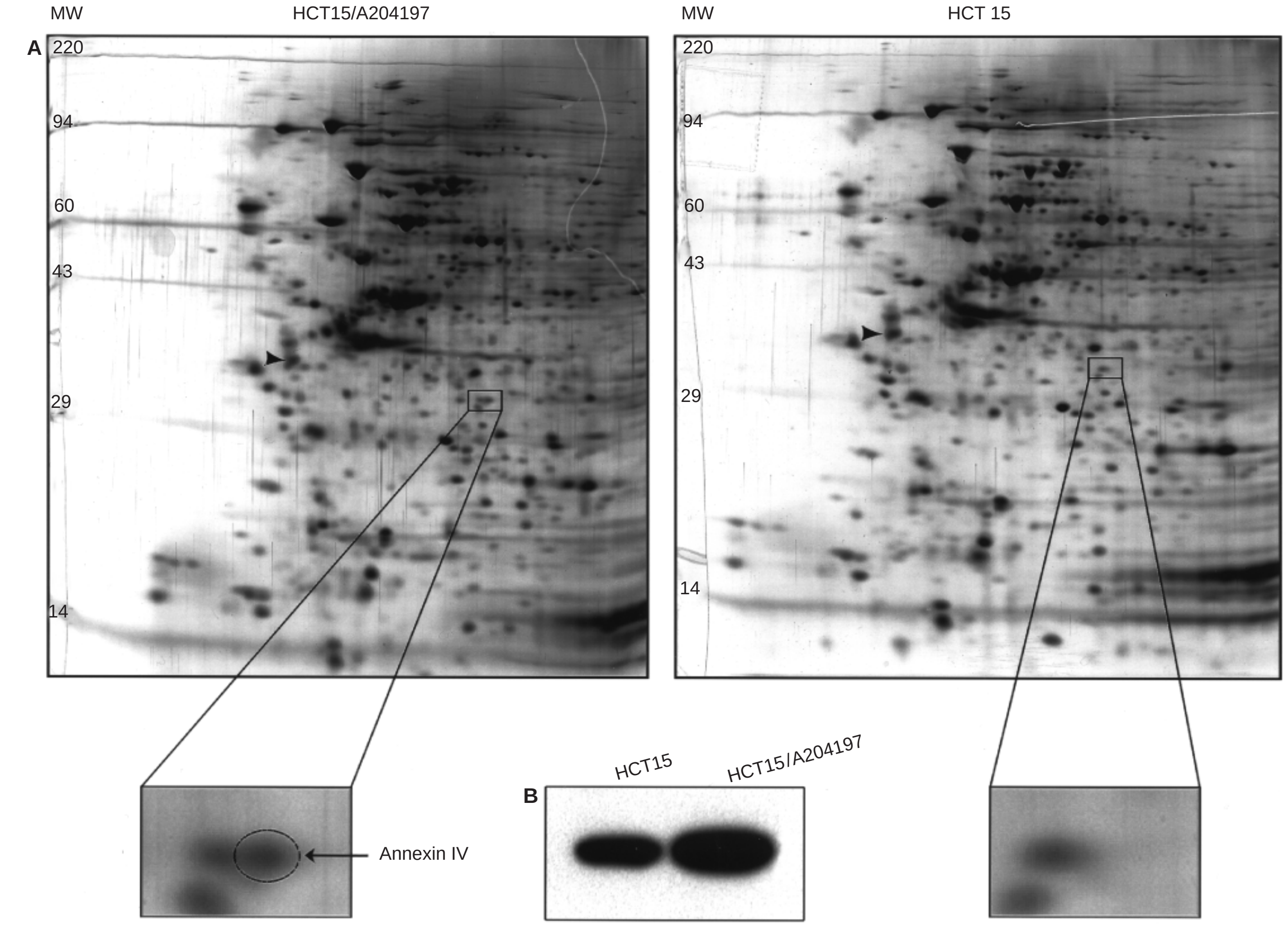

**Figure 1** **(A)** Two-dimensional gel mapping of HCT15 and HCT15/A204197 cells. Acidic proteins are oriented to the left, basic to the right, low molecular weight proteins toward the bottom (MW in kilodaltons), and high MW proteins toward the top. The horizontal dimension represents a pH gradient of 4–8. The arrowhead indicates the internal standard, tropomyosin (MW 33 kDa, pI = 5.2). Annexin IV protein (calculated pI = 6.4, MW = 30 kDa) was indicated by circle in HCT15/A204197 cells. (**B**) Western blot analysis of annexin IV in HCT15 and HCT15/A204197. For details see Materials and Methods generated A204197-resistant HCT15 cells by increasing stepwise concentration of A204197. A204197-resistant HCT15 cells (designated as HCT15/A204197) were maintained in 200 nM A204197 and displayed a 4.2-fold increase in resistance to A204197 when compared to the parental HCT15 cells. The $IC_{50}$ value increased from 40.8 nM to 172 nM. HCT15/A204197 cells also showed slight increase in resistance to other antimitotic compounds such as paclitaxel, colcemid or nocodazole (data not shown). To examine if any proteins are overexpressed or altered in HCT15/A204197 cells, we performed two-dimensional gel mapping analysis. As shown in Figure 1A, there was one spot (calculated MW ~30 kDa, pI ~6.0) which was overexpressed in HCT15/A204197 cells. Computer analysis of the two-dimensional gel showed that this was the most distinct spot to be overexpressed in A204197 resistant HCT15 cells. This spot was cut out and mass spectroscopic analysis was done following trypsin digestion. Partial protein sequence determination of trypsin digested fragments revealed that the unknown protein was annexin IV. To further confirm that the annexin IV protein is overexpressed in HCT15/A204197 cells, Western blot analysis was performed in HCT15/A204197 cells. As shown in Figure 1B, annexin IV protein was overexpressed in HCT15/A204197 cells by approximately twofold. Since we know more about paclitaxel-resistant H460 cells and given paclitaxel's importance as a clinical anticancer therapeutic, we decided to focus on these cells for our annexin IV study.

### Annexin IV overexpression in paclitaxel-resistant H460 cell line

It was previously shown that annexin II expression was elevated in doxorubicin selected MDR small-cell lung cancer cell line (Cole et al, 1992). We further examined whether annexin II or annexin IV was overexpressed in paclitaxel-resistant variants derived from H460 cells. Previously we developed a series of paclitaxel-resistant cell lines from H460 lung cancer cells to study the mechanism of paxlitaxel resistance. For example, the H460/T800 cell line was maintained at 800 nM paclitaxel and was over 1000-fold resistant to paclitaxel. This cell line overexpresses P-gp and exhibits cross-resistance to other MDR compounds (characterization of paclitaxel-resistant H460 cell lines will be published elsewhere). Cell extracts from each selection step (T100, T200, T400, T800; T stands for paclitaxel and each number represents nM concentration of paclitaxel maintained in the medium) were analysed for annexin II and annexin IV expression by Western blot analysis. As shown in Figure 2A, as cells became more resistant to paclitaxel there was a corresponding increase in expression of annexin IV protein.

*© 2000 Cancer Research Campaign*

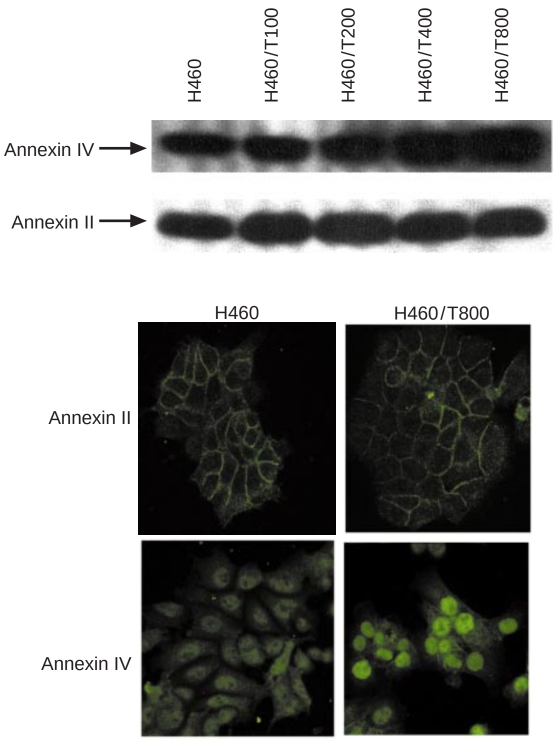

**Figure 2** Overexpression of annexin IV in paclitaxel-resistant H460 cells. (**A**) Cell extracts from paclitaxel-resistant cell lines were prepared and analysed on SDS-PAGE then transferred to PVDF membrane. The membrane was probed with either annexin IV or annexin II antibody. (**B**) Immunostaining using either annexin IV or annexin II was performed in H460 and H460/T800 cells. For details see Materials and Methods.

However, annexin II protein expression was not altered in paclitaxel-resistant cell lines.

To further confirm and localize the expression of annexin IV and II in H460/T800 cells, we performed immnohistochemistry using either annexin IV or annexin II antibody. As shown in Figure 2B, there was an intense staining of annexin IV in the nucleus of H460/T800 cells. In addition a diffuse staining of annexin IV was observed in the cytosol of H460/T800 cells. In contrast, a diffused annexin IV staining was seen in both the cytosol and nucleus in H460 parental cells. There was no difference in annexin II staining in either control H460 or H460/T800 cells. Annexin II staining was localized to the membrane in both H460 and H460/T800 cells.

### Short-term effects of paclitaxel on annexin IV expression in H460/T800 cells

To examine whether short-term treatment with paclitaxel can induce annexin IV protein expression, exponentially growing H460 cells were treated with 10 nM paclitaxel and the expression of annexin IV was determined over a 4-day period. As shown in Figure 3A, there was a gradual increase in annexin IV protein during the 4-day period. However, annexin II protein expression was not affected during the same period.

We examined the change in annexin IV protein by immunohistochemistry during the 4-day paclitaxel treatment in H460 cells. As shown in Figure 3B, there was an increase in fluorescence intensity of annexin IV in the nucleus in 10nM paclitaxel-treated cells starting at day 1 and compared to the untreated controls. The intensity was stronger at day 4. The increase in signal was not homogeneous for the population as a whole. However, overall annexin IV staining in paclitaxel-treated cells was much stronger than the control cell line.

To determine whether annexin IV expression has any correlation with paclitaxel resistance following 4-day paclitaxel treatment, cells were analysed for cell proliferation in the presence of increasing concentrations of paclitaxel. As shown in Figure 3C, pretreatment with paclitaxel led to ~fivefold increase in the cell proliferation $IC_{50}$ when compared to the control cell line (27 nM versus 5.2 nM respectively). During this time period, there was no induction of P-gp or β-tubulin as determined by Western blot analysis (data not shown).

### Transfection of annexin IV cDNA into 293T cells

To further confirm that annexin IV is involved in paclitaxel resistance, we transfected annexin IV cDNA into 293T cells. Following transfection cells were selected with G418 (1 mg $ml^{-1}$) and a number of G418-resistant clones were obtained and analysed. As shown in Figure 4A, two annexin IV clones (#9, #12) overexpressed at least twofold more annexin IV protein than the vector clones (Vt#15, Vt#18). Immunostaining study showed that annexin IV clones display stronger nuclear staining for annexin IV (data not shown). These clones were analysed for cell proliferation in the presence of drugs using SRB assay. As shown in Figure 4B, annexin IV clones displayed a ~threefold increase in the $IC_{50}$ against paclitaxel. Similarly, there was a 1.5-fold increase in the $IC_{50}$ of annexin IV clones against colchicine or nocodazole when compared to the vector control clones.

### DISCUSSION

In this report, we have demonstrated that:

1. overexpression of annexin IV was identified in HCT15/A204197 cells by two-dimensional gel protein mapping, mass spectroscopy and confirmed by Western blot analysis
2 overexpression of annexin IV was also seen in paclitaxel-resistant cells derived from H460 cell line as determined by both Western blot analysis and immunostaining
3. acute treatment of H460 cells with 10 nM paclitaxel resulted in induction of annexin IV and conferred ~ fivefold increase in resistance to paclitaxel
4. transfection of annexin IV cDNA into 293T cells conferred threefold increase in resistance to paclitaxel. To our knowledge, this is the first report demonstrating that annexin IV plays a role in paclitaxel resistance.

Annexin IV belongs to a multigene family of annexins, which bind to phopholipids in a calcium-dependent manner (Smith and Moss, 1994). Although the role for each member of the annexin family is not well understood, annexins seem to be involved in several cellular processes such as exocytosis, endocytosis and ion channel activity (Smith and Moss, 1994). Our results suggest that annexin IV may be one of the proteins to be induced in cells under stress conditions such as antimitotic drug exposure. Previously another annexin family member, annexin II, was shown to be overexpressed in a doxorubicin-resistant small-lung cancer cell line, H69AR (Cole et al, 1992). However, in our study, annexin II was not induced in both paclitaxel-resistant H460 cells and H460 cells

 © 2000 Cancer Research Campaign

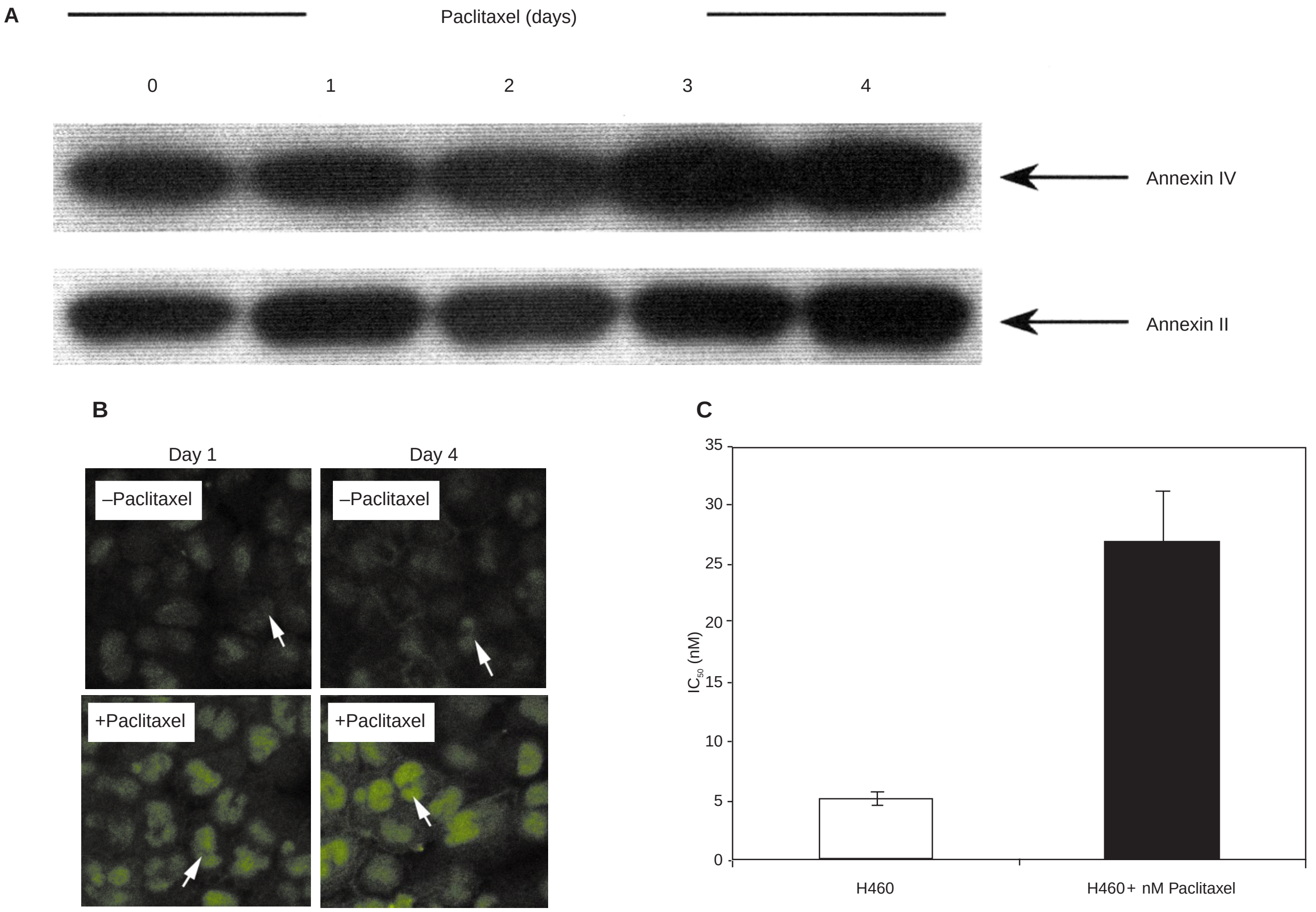

**Figure 3** Acute treatment of H460 cells with 10 nM paclitaxel upregulates annexin IV. (**A**) Cell extracts of H460 cells treated with 10nM paclitaxel were collected up to 4 days. Western blot was performed using either annexin IV or annexin II antibody as described in Figure 2 and Materials and Methods. (**B**) Immunostaining of annexin IV in H460 cells treated with or without 10 nM paclitaxel for 1 or 4 days. Arrows indicate the nucleus. (**C**) H460 cells pretreated with 10 nM paclitaxel for 4 days were used in $IC_{50}$ determination of paclitaxel by SRB assay 2 days later. The $IC_{50}$s of paclitaxel for H460 cells was 5.2 ± 0.5 nM, whereas 10 nM paclitaxel-treated H460 cells was 27 ± 4.0 nM

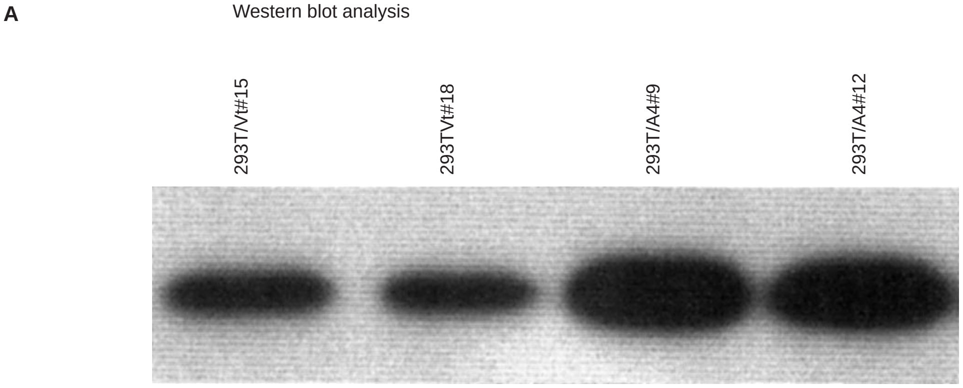

**B** Determination of $IC_{50}$

| | Vt#15 | Vt#18 | Mean | A4#9 | A4#12 | Mean |
|---|---|---|---|---|---|---|
| Paclitaxel | 80.9 | 94.9 | 87.9 | 265.3 | 237.3 | 251.3 |
| Colchicine | 30.3 | 23.4 | 26.9 | 31.9 | 47.8 | 39.9 |
| Nocodazole | 336 | 169.8 | 252.9 | 336.5 | 432.5 | 384.5 |

**Figure 4** Annexin IV overexpressing clones confer resistance to paclitaxel. (**A**) Western blot analysis of two vector (293T/Vt#15, 293T/Vt#18) and two annexin IV (293T/A4#9, 293T/A4#12) clones for annexin IV protein expression. (**B**) $IC_{50}$ determination of annexin IV clones by SRB assay. For details see Materials and Methods

*© 2000 Cancer Research Campaign* 

treated with paclitaxel. Thus it is possible that induction of different classes of annexins by cytotoxic drugs might be cell type-specific. Alternatively, different cytotoxic drugs may induce individual classes of annexin proteins.

How does annexin IV contribute to paclitaxel resistance? We do not yet know the mechanism by which annexin IV induces paclitaxel resistance. It is possible that annexin IV may interact with other signalling proteins that may influence the paclitaxel-induced drug resistance. Alternatively, it has been shown that MDR cells display elevated levels of cytoplasmic calcium (Nygren et al, 1991). Acute paclitaxel treatment increases the calcium level in H460 cells as determined by using the calcium-binding indicator Indo-1 AM (data not shown). Previously, an increase in calcium levels in cells have been shown to induce translocation of annexin IV in several human cell lines (Sjolin et al, 1994; Mohiti et al, 1995; Barwise and Walker, 1996; Raynal et al, 1996). Thus in H460 cells or paclitaxel-resistant H460 cells, paclitaxel induced changes in the intracellular calcium levels and this in turn may have induced annexin IV expression and its translocation. Whether the overexpression of annexin IV in H460/T800 cells is due to amplification of the annexin IV gene is unknown.

Several reports show that paclitaxel resistance is due to a number of factors besides P-gp. For example, β-tubulin mutation and alterations have been shown to be associated with paclitaxel resistance (Ranganathan et al, 1988, 1996, 1998; Haber et al, 1995; Giannakakou et al, 1997; Kavallaris et al, 1997, 1999). We have shown that α-tubulin also contributes paclitaxel resistance (Han et al, 1999). Others have shown that caveolin I is up-regulated in paclitaxel-resistant cell lines (Yang et al, 1998). So far we have examined only annexin II and annexin IV expression in paclitaxel-resistant cells and we are currently examining other classes of annexins and the role they may play in drug resistance.

Here we have shown that paclitaxel induces expression of annexin IV, but not annexin II and annexin IV plays a role in paclitaxel resistance. Furthermore, annexin IV alone can confer paclitaxel resistance. Therefore, modulation of annexin IV may be a novel therapeutic target for cells or tumours that have high level of annexin IV.

### REFERENCES

Barwise JL and Walker JH (1996) Annexins II, IV, V and VI relocate in response to rises in intracellular calcium in human foreskin fibroblasts. *J Cell Sci* **109**: 247–255

Benz J and Hofmann A (1997) Annexins: from structure to function. *Biol Chem* **78**: 177–183

Blagosklonny MV, Schulte TW, Nguyen P, Mimnaugh EG, Trepel J and Neckers L (1995) Taxol induction of p21WAF1 and p53 requires c-raf-1. *Cancer Res* **55**: 4623–4626

Cabral F, Abraham I and Gottesman MM (1981) Isolation of a taxol-resistant Chinese hamster ovary cell mutant that has an alteration in α-tubulin. *Proc Natl Acad Sci USA* **78**: 4388–4391

Cole SPC, Pinkoski MJ, Bhardwaj G and Deeley RG (1992) Elevated expression of annexin II (lipocortin II, p36) in a multidrug resistant small cell lung cancer cell line. *Br J Cancer* **65**: 498–502

Dubois T, Oudinet JP, Mira JP and Russo-Marie F (1996) Annexins and protein kinase C. *Biochim Biophys Acta* **1313**: 290–294

Germann UA (1996) P-glycoprotein-a mediator of multidrug resistance in tumour cells. *Eur J Cancer* **32A**: 927–944

Giannakakou P, Sackett DL, Kang YK, Zhan Z, Buters JTM, Fojo T and Poruchynsky MS (1997) Paclitaxel-resistant human ovarian cancer cells have mutant β-tubulins that exhibit impaired paclitaxel-driven polymerization. *J Biol Chem* **272**: 17118–17125

Gottesman MM and Pastan I (1988) The multidrug transporter, a double-edged sword. *J Biol Chem* **263**: 12163–12166

Gottesman MM and Pastan I (1993) Biochemistry of multidrug resistance mediated by the multidrug transporter. *Annu Rev Biochem* **62**: 385–427

Haber M, Burkhart CA, Regl DL, Madafiglio J and Norris MD and Horwitz SB (1995) Altered expression of Mβ2, the class II β-tubulin isotype, in a murine J744.2 cell line with a high level of taxol resistance. *J Biol Chem* **270**: 31269–31277

Han EK-H, Sgambato A, Jiang W, Zhang Y-U, Santella RM, Doki Y, Cacace A, Schieren I and Weinstein IB (1995) Stable overexpression of cyclin D1 in a human mammary epithelial cell line prolongs the S-phase and inhibits growth. *Oncogene* **10**: 953–961

Han EK-H, Begemann M, Sgambato A, Soh J-W, Xing W-Q and Weinstein IB (1996) Increased expression of cyclin D1 in a murine mammary epithelial cell line inhibits growth and enhances apoptosis. *Cell Growth Differ* **7**: 699–710

Han EK-H, Gehrke L, Tahir SK, Credo RB, Cherian SP, Sham H, Rosenberg SH and Ng SC (1999) Modulation of multidrug resistance by α-tubulin in taxol resistant human lung cancer cell line. *Eur J Cancer* (in press)

Kavallaris M, Kuo DY-S, Burkhart CA, Regl DL, Norris MD, Haber M and Horwitz SB (1997) Taxol-resistant epithelial ovarian tumors are associated with altered expression of specific β-tubulin isotypes. *J Clin Invest* **100**: 1282–1293

Kavallaris M, Burkhart CA and Horwitz SB (1999) Antisense oligonuclotides to class III β-tubulin sensitize drug-resistant cells to taxol. *Br J Cancer* **80**: 1020–1025

Lee JS, Paull K, Alvarez M, Hose C, Monks A, Grever M, Fojo AT and Bates SE (1994) Rhodamine efflux patterns predict P-glycoprotein substrates in the National Cancer Institute drug screen. *Mol Pharm* **46**: 627–638

Lehnert M (1996) Clinical multidrug resistance in cancer: a multifactorial problem. *Eur J Cancer* **32A**: 912–920

Mohiti J, Caswell AM and Walker JH (1995) Calcium-induced relocation of annexins IV and V in the human osteosarcoma cell line MG-63. *Mol Mem Biol* **12**: 321–329

Nygren P, Larsson R, Gruber A, Peterson C and Bergh J (1991) Doxorubicin selected multidrug-resistant small cell lung cancer cell lines characterized by elevated cytoplasmic Ca2+ and resistance modulation by verapamil in absence of P-glycoprotein overexpression. *Br J Cancer* **64**: 1011–1018

O'Farrell PH (1975) High resolution two-dimensional electrophoresis of proteins. *J Biol Chem* **250**: 4007–4021

Ranganathan S, Dexter DW, Benetatos CA and Hudes GR (1988) Cloning and sequencing of human βIII-tubulin cDNA: induction of βIII isotype in human prostate carcinoma cells by acute exposure to antimicrotubule agents. *Biochim Biophy Acta* **1395**: 237–245

Ranganathan S, Dexter DW, Benetatos CA, Chapman AE, Tew KD and Hudes GR (1996) Increase of βIII- and βIV-tubulin isotypes in human prostate carcinoma cells as a result of estramustine resistance. *Cancer Res* **56**: 2584–2589

Ranganathan S, Benetatos CA, Colarusso PJ, Dexter DW and Hudes GR (1998) Altered β-tubulin isotype expression in paclitaxel-resistant human prostate carcinoma cells. *Br J Cancer* **77**: 562–566

Raynal P, Kuijpers G, Rojas E and Pollard HB (1996) A rise in nuclear calcium translocate annexins IV and V to the nuclear envelope. *FEBS Lett* **392**: 263–268

Rowinsky EK and Donehower RC (1995) Paclitaxel (Taxol) *N Engl J Med* (1995) **332**: 1004–1014

Rowinsky EK, Onetto N, Canetta RM and Arbuck SG (1992) Taxol: the first of the taxanes, an important new class of antitumor agents. *Sem Oncol* **19**: 646–662

Sjolin C, Stendahl O and Dahlgren C (1994) Calcium-induced translocation of annexins to subcellular organelles of human neutrophils. *Biochem J* **300**: 325–330

Schibler MJ and Cabral F (1986) Taxol-dependent mutants of Chinese hamster ovary cells with alterations in alpha-and beta-tubulin. *J Cell Biol* **102**: 1522–1531

Skehan P, Storeng R, Scudiero D, Monks A, McMahon J, Vistica D, Warren JT, Bokesch H, Keney S and Boyd MR (1990) New colorimetric cytotoxicity assay for anticancer-drug screening. *J Natl Cancer Inst* **82**: 1107–1112

Smith PD and Moss SE (1994) Structural evoluation of the annexin supergene family. *Trends in Genetics* **10**: 241–246

Wang TH, Wang HS, Ichijo H, Giannakakou P, Foster JS, Fojo T and Wimalasena J (1998) Microtubule-interfering agents activate c-Jun N-terminal kinase/stress-activated protein kinase through both Ras and apoptosis signal-regulating kinase pathways. *J Biol Chem* **273**: 4928–4936

Wolfson M, Yang CPH and Horwitz SB (1997) Taxol induces tyrosine phosphorylation of Shc and its association with Grb2 in murine RAW 264.7 cells. *Int J Cancer* **70**: 248–252

Yang CPH, Galbiati F, Volonte D, Horwitz SB and Lisanti MP (1998) Upregulation of caveolin-1 and caveola organelles in taxol-resistant A549 cells. *FEBS Lett* **439**: 368–372

 © 2000 Cancer Research Campaign

## References

[PDF_00480] Barwise J. L., Walker J. H. (1996). Annexins II, IV, V and VI relocate in response to rises in intracellular calcium in human foreskin fibroblasts.. J Cell Sci.

[PDF_00483] Benz J., Hofmann A. (1997). Annexins: from structure to function.. Biol Chem.

[PDF_00485] Blagosklonny M. V., Schulte T. W., Nguyen P., Mimnaugh E. G., Trepel J., Neckers L. (1995). Taxol induction of p21WAF1 and p53 requires c-raf-1.. Cancer Res.

[PDF_00488] Cabral F., Abraham I., Gottesman M. M. (1981). Isolation of a taxol-resistant Chinese hamster ovary cell mutant that has an alteration in alpha-tubulin.. Proc Natl Acad Sci U S A.

[PDF_00491] Cole S. P., Pinkoski M. J., Bhardwaj G., Deeley R. G. (1992). Elevated expression of annexin II (lipocortin II, p36) in a multidrug resistant small cell lung cancer cell line.. Br J Cancer.

[PDF_00494] Dubois T., Oudinet J. P., Mira J. P., Russo-Marie F. (1996). Annexins and protein kinases C.. Biochim Biophys Acta.

[PDF_00496] Germann U. A. (1996). P-glycoprotein--a mediator of multidrug resistance in tumour cells.. Eur J Cancer.

[PDF_00498] Giannakakou P., Sackett D. L., Kang Y. K., Zhan Z., Buters J. T., Fojo T., Poruchynsky M. S. (1997). Paclitaxel-resistant human ovarian cancer cells have mutant beta-tubulins that exhibit impaired paclitaxel-driven polymerization.. J Biol Chem.

[PDF_00504] Gottesman M. M., Pastan I. (1993). Biochemistry of multidrug resistance mediated by the multidrug transporter.. Annu Rev Biochem.

[PDF_00502] Gottesman M. M., Pastan I. (1988). The multidrug transporter, a double-edged sword.. J Biol Chem.

[PDF_00506] Haber M., Burkhart C. A., Regl D. L., Madafiglio J., Norris M. D., Horwitz S. B. (1995). Altered expression of M beta 2, the class II beta-tubulin isotype, in a murine J774.2 cell line with a high level of taxol resistance.. J Biol Chem.

[PDF_00514] Han E. K., Begemann M., Sgambato A., Soh J. W., Doki Y., Xing W. Q., Liu W., Weinstein I. B. (1996). Increased expression of cyclin D1 in a murine mammary epithelial cell line induces p27kip1, inhibits growth, and enhances apoptosis.. Cell Growth Differ.

[PDF_00510] Han E. K., Sgambato A., Jiang W., Zhang Y. J., Santella R. M., Doki Y., Cacace A. M., Schieren I., Weinstein I. B. (1995). Stable overexpression of cyclin D1 in a human mammary epithelial cell line prolongs the S-phase and inhibits growth.. Oncogene.

[PDF_00523] Kavallaris M., Burkhart C. A., Horwitz S. B. (1999). Antisense oligonucleotides to class III beta-tubulin sensitize drug-resistant cells to Taxol.. Br J Cancer.

[PDF_00520] Kavallaris M., Kuo D. Y., Burkhart C. A., Regl D. L., Norris M. D., Haber M., Horwitz S. B. (1997). Taxol-resistant epithelial ovarian tumors are associated with altered expression of specific beta-tubulin isotypes.. J Clin Invest.

[PDF_00526] Lee J. S., Paull K., Alvarez M., Hose C., Monks A., Grever M., Fojo A. T., Bates S. E. (1994). Rhodamine efflux patterns predict P-glycoprotein substrates in the National Cancer Institute drug screen.. Mol Pharmacol.

[PDF_00529] Lehnert M. (1996). Clinical multidrug resistance in cancer: a multifactorial problem.. Eur J Cancer.

[PDF_00531] Mohiti J., Caswell A. M., Walker J. H. (1995). Calcium-induced relocation of annexins IV and V in the human osteosarcoma cell line MG-63.. Mol Membr Biol.

[PDF_00534] Nygren P., Larsson R., Gruber A., Peterson C., Bergh J. (1991). Doxorubicin selected multidrug-resistant small cell lung cancer cell lines characterised by elevated cytoplasmic Ca2+ and resistance modulation by verapamil in absence of P-glycoprotein overexpression.. Br J Cancer.

[PDF_00538] O'Farrell P. H. (1975). High resolution two-dimensional electrophoresis of proteins.. J Biol Chem.

[PDF_00547] Ranganathan S., Benetatos C. A., Colarusso P. J., Dexter D. W., Hudes G. R. (1998). Altered beta-tubulin isotype expression in paclitaxel-resistant human prostate carcinoma cells.. Br J Cancer.

[PDF_00544] Ranganathan S., Dexter D. W., Benetatos C. A., Chapman A. E., Tew K. D., Hudes G. R. (1996). Increase of beta(III)- and beta(IVa)-tubulin isotopes in human prostate carcinoma cells as a result of estramustine resistance.. Cancer Res.

[PDF_00540] Ranganathan S., Dexter D. W., Benetatos C. A., Hudes G. R. (1998). Cloning and sequencing of human betaIII-tubulin cDNA: induction of betaIII isotype in human prostate carcinoma cells by acute exposure to antimicrotubule agents.. Biochim Biophys Acta.

[PDF_00550] Raynal P., Kuijpers G., Rojas E., Pollard H. B. (1996). A rise in nuclear calcium translocates annexins IV and V to the nuclear envelope.. FEBS Lett.

[PDF_00553] Rowinsky E. K., Donehower R. C. (1995). Paclitaxel (taxol). N Engl J Med.

[PDF_00555] Rowinsky E. K., Onetto N., Canetta R. M., Arbuck S. G. (1992). Taxol: the first of the taxanes, an important new class of antitumor agents.. Semin Oncol.

[PDF_00561] Schibler M. J., Cabral F. (1986). Taxol-dependent mutants of Chinese hamster ovary cells with alterations in alpha- and beta-tubulin.. J Cell Biol.

[PDF_00558] Sjölin C., Stendahl O., Dahlgren C. (1994). Calcium-induced translocation of annexins to subcellular organelles of human neutrophils.. Biochem J.

[PDF_00563] Skehan P., Storeng R., Scudiero D., Monks A., McMahon J., Vistica D., Warren J. T., Bokesch H., Kenney S., Boyd M. R. (1990). New colorimetric cytotoxicity assay for anticancer-drug screening.. J Natl Cancer Inst.

[PDF_00566] Smith P. D., Moss S. E. (1994). Structural evolution of the annexin supergene family.. Trends Genet.

[PDF_00568] Wang T. H., Wang H. S., Ichijo H., Giannakakou P., Foster J. S., Fojo T., Wimalasena J. (1998). Microtubule-interfering agents activate c-Jun N-terminal kinase/stress-activated protein kinase through both Ras and apoptosis signal-regulating kinase pathways.. J Biol Chem.

[PDF_00572] Wolfson M., Yang C. P., Horwitz S. B. (1997). Taxol induces tyrosine phosphorylation of Shc and its association with Grb2 in murine RAW 264.7 cells.. Int J Cancer.

[PDF_00575] Yang C. P., Galbiati F., Volonte D., Horwitz S. B., Lisanti M. P. (1998). Upregulation of caveolin-1 and caveolae organelles in Taxol-resistant A549 cells.. FEBS Lett.

